# A new karyotype for *Rhipidomys* (Rodentia, Cricetidae) from Southeastern Brazil

**DOI:** 10.3897/CompCytogen.v6i3.2432

**Published:** 2012-07-09

**Authors:** Ana Heloisa de Carvalho, Maria Olímpia Garcia Lopes, Marta Svartman

**Affiliations:** 1 Universidade Federal de Minas Gerais, Laboratório de Citogenética Evolutiva, Departamento de Biologia Geral, Instituto de Ciências Biológicas, Av. Antônio Carlos, 6627 – Pampulha, 31270-910, Belo Horizonte, Brazil; 2 Biodiversity Salvation, Rua João Pinheiro, 282, Centro, 37925-000, Piumhi, Minas Gerais, Brazil

**Keywords:** *Rhipidomys*, chromosome banding, FISH

## Abstract

In this work we present a new karyotype for *Rhipidomys* Tschudi, 1845 (Cricetidae, Rodentia) from Brazil. Our chromosome analyses included GTG- and CBG-banding patterns, the localization of the nucleolus organizer regions after silver staining (Ag-NORs) and fluorescence *in situ* hybridization (FISH) with a telomere probe. The new karyotype is composed of 44 chromosomes and has a fundamental number (number of autosomal arms) of 48. Most *Rhipidomys* species already karyotyped presented similar complements with 2n=44, but their fundamental numbers varied from FN=46 to 80, a variation that has been mainly attributed to pericentric inversions. The comparison of this new karyotype to those of other *Rhipidomys* already reported allowed us to conclude that it is a distinctive chromosome complement, which can be of great use as a tool for the very complicated taxonomic identification in this genus.

## Introduction

The Neotropical rodent *Rhipidomys* Tschudi, 1845 (family Cricetidae) is an arboreal genus belonging to the largely diverse subfamily Sigmodontinae, whose phylogenetic relationships are difficult to resolve, resulting in taxonomic uncertainties at every level, from species to tribes (Musser and Carleton 2005).

*Rhipidomys* is widely distributed and has been reported from Panama to southeastern Brazil and northern Argentina. The distribution of many species remains uncertain and there are several reports of undescribed species (Tribe 1996, Musser and Carleton 2005). Besides the eighteen species recognized by Musser and Carleton (2005), three additional species have been identified: *Rhipidomys ipukensis* Rocha et al., 2011, *Rhipidomys tribei* Costa et al., 2011 and *Rhipidomys itoan* Costa et al., 2011, and a further unnamed clade from central and eastern Brazil has been recognized (Costa et al. 2011, Rocha et al. 2011). Twelve of the recognized *Rhipidomys* species have been found in Brazil: *Rhipidomys macconnelli* De Winton, 1900, *Rhipidomys leucodactylus* Tschudi, 1845, *Rhipidomys wetzeli* Gardner, 1989, *Rhipidomys nitela* Thomas, 1901, *Rhipidomys macrurus* Gervais, 1855, *Rhipidomys gardneri* Patton et al., 2000, *Rhipidomys emiliae* J.A. Allen, 1916, *Rhipidomys mastacalis* Lund, 1840, *Rhipidomys cariri* Tribe, 2005, *Rhipidomys ipukensis*, *Rhipidomys tribei* and *Rhipidomys itoan*, and an additional undescribed species has been reported as *Rhipidomys* sp 2 (Tribe 1996, Musser and Carleton 2005, Bonvicino et al. 2008, Costa et al. 2011).

Eleven species of *Rhipidomys* have already been karyotyped and, with the exception of *Rhipidomys nitela* (2n=48) and *Rhipidomys* prope *nitela* (2n=50), all presented karyotypes with 2n=44 chromosomes. In contrast with the conservation of diploid numbers, the karyotypes of *Rhipidomys* showed fundamental numbers ranging from FN=46 to 80, a variation mainly attributed to pericentric inversions. The available karyotypical data for *Rhipidomys* are summarized in Table 1. Most cytogenetic studies on this genus were performed with conventionally stained karyotypes and in less than half the GTG- CBG- or AgNOR-banding patterns were also included.

**Table:** **Table 1.** Summary of the available chromosome data for *Rhipidomys*.^1^ As *Rhipidomys sclateri*, which was later considered a synonym of *Rhipidomys leucodactylus* (Musser and Carleton 2005). ^2^ Identified by Tribe (1996), originally reported as *Rhipidomys* sp. ^3^ As *Rhipidomys cearanus* (Zanchin et al. 1992), later considered as a synonym of *Rhipidomys mastacalis* (Musser and Carleton 2005).

**Group**	**Species**	**2n/FN**	**Locality**	**Reference**
*Rhipidomys leucodactylus*	*Rhipidomys leucodactylus*	44/ 46	Rio Juruá (AM)	Patton et al. 2000
		44/ 48	Rio Jamari (RO), Caldas Novas, Serra da Mesa (GO)	Zanchin et al. 1992, Andrades-Miranda et al. 2002
		44/ 48^1^	Cueva del Agua (Venezuela)	Aguilera et al. 1994
		44/ 52	Serra da Mesa (GO), Caxiuanã (PA)	Andrades-Miranda et al. 2002
	*Rhipidomus* sp.	44/ 48	Berilo (MG)	This work
	*Rhipidomys latimanus*	44/ 48	Peñas Blancas (Colômbia)	Gardner and Patton 1976
	*Rhipidomys macrurus*	44/ 48	Águas Emendadas (DF), Chapada Diamantina (BA)	Svartman and Almeida 1993, Pereira and Geise 2007
		44/ 49	Granja do Ipê (DF)	Svartman and Almeida 1993
	*Rhipidomys* prope *macrurus*	44/ 49^2^	Casa Grande (SP)	Svartman and Almeida 1993
		44/ 50^2^	Monte Verde (ES)	Zanchin et al. 1992
		44/ 50	Garrafão (RJ)	Tribe 1996
		44/ 51	Mocambinho (MG)	Tribe 1996
	*Rhipidomys gardneri*	44/ 50	Rio Juruá (AC)	Patton et al. 2000
	*Rhipidomys macconnelli*	44/ 50	La Escalera (Venezuela)	Aguilera et al. 1994
	*Rhipidomys cf. mastacalis*	44/ 52	Vila Rica (MT), Aripuanã (MT)	Silva and Yonenaga-Yassuda 1999
	*Rhipidomys itoan*	44/ 48,49,50	SP and RJ	Costa et al. 2011
*Rhipidomys mastacalis*	*Rhipidomys mastacalis*	44/ 74	Lagoa Santa (MG), Unacau (BA), Casimiro de Abreu (RJ), Reserva Biológica Duas Bocas (ES)	Zanchin et al. 1992, Paresque et al. 1994, Tribe 1996
		44/ 76	Serra da Mesa (GO)	Andrades-Miranda et al. 2002
		44/ 80	Serra da Mesa (GO)	Andrades-Miranda et al. 2002
		^3^44/ high	Serra dos Cavalos (PE)	Zanchin et al. 1992
Hybrid	*Rhipidomys* with high FN x *Rhipidomys* with low FN	44/ 61	M. Chapéu (BA)	Silva and Yonenaga-Yassuda 1999
*Rhipidomys nitela*	*Rhipidomys nitela*	44/ 71	San Ignacio, (Venezuela)	Tribe 1996
		48/ 67	La Trinité (French Guiana)	Volobouev and Catzeflis 2000
		48/ 68	Surumurú (RR)	Andrades-Miranda et al. 2002
	*Rhipidomys* prope *nitela*	50/ 71,72	Manaus (AM)	Silva and Yonenaga-Yassuda 1999

In this work, we present a new karyotype for *Rhipidomys*. Our analyses included GTG- and CBG-banding patterns, the silver staining location of the nucleolus organizer regions (Ag-NORs) and fluorescence *in situ* hybridization (FISH) with a telomere probe.

## Material and methods

**Figure 1.**
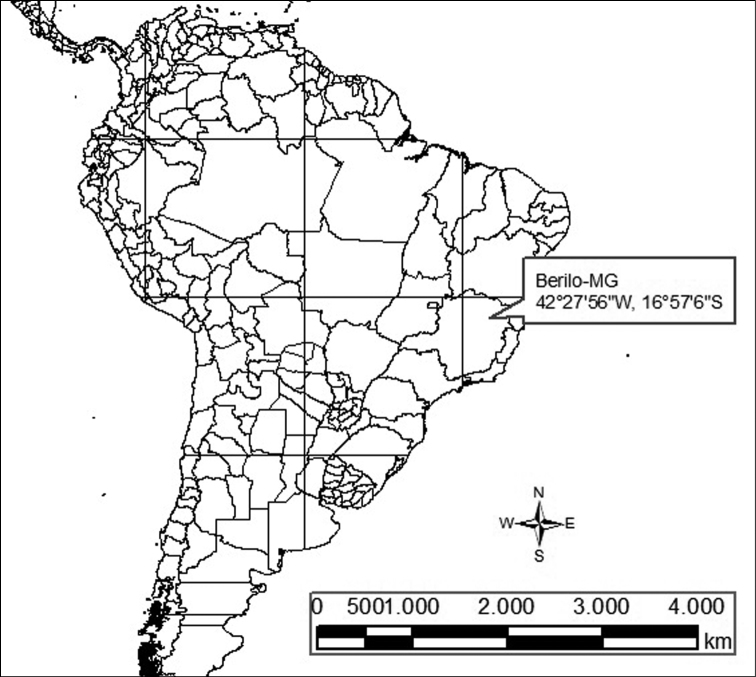
Map showing the collection locality of the *Rhipidomys* sp. analyzed.

We analyzed five specimens (two males and three females) of *Rhipidomys* sp. captured in 2004 in a dry land region in the margins of the Jequitinhonha river, in Berilo, state of Minas Gerais, Brazil (16°57'06"S, 42°27'56"W; Fig. 1) under the license 129/04-NUFAS/MG from the Instituto Brasileiro para o Meio Ambiente - IBAMA. The skins and skulls were deposited at the Museu de Ciências Naturais da Pontifícia Universidade Católica de Minas Gerais, in Belo Horizonte, Minas Gerais State, Brazil, under the numbers: MCNM 1643, 1644 (two males) and MCNM 1646, 1647, 1648 (three females).

Chromosome preparations were obtained from bone marrow according to the technique described by Ford and Hamerton (1956). GTG- and CBG-banding patterns were obtained following Seabright (1971) and Sumner (1972), respectively, and silver staining of the nucleolus organizer regions (Ag-NORs) was performed according to Howell and Black (1980). FISH with the (T_2_AG_3_)_n_ telomere sequence was performed with the Telomere PNA Kit/FITC (Dako Cytomation) according to the manufacturer’s instructions.

The chromosomes were arranged based on the karyotype described for specimens of *Rhipidomys* sp. by Svartman and Almeida (1993), which were later identified as *Rhipidomys macrurus* (Tribe 1996).

## Results

**Figure 2.**
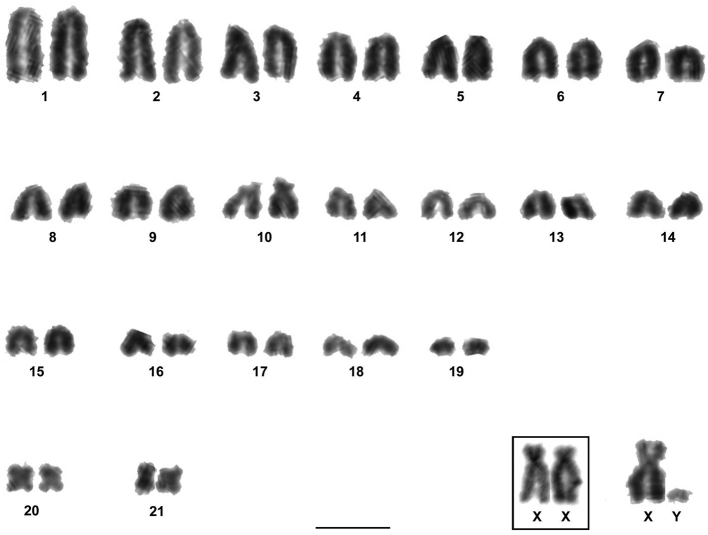
Karyotype of *Rhipidomys* sp. male (2n=44, FN=48) after conventional Giemsa staining. In the inset, the sex chromosomes of a female. Note the variation in the size of the short arms of the X chromosomes. Bar = 10 µm.

The two males and three females of *Rhipidomys* sp. analyzed presented a diploid number of 2n=44 chromosomes and a fundamental number FN=48. This karyotype was composed of 21 pairs of autosomes: 18 pairs of acrocentrics with gradual variation in size from large to small (pairs 1 to 9 and 11 to 19), one pair of medium subtelocentrics (pair 10), one pair of small metacentrics (pair 20) and one pair of small submetacentrics (pair 21). The X chromosome was a large submetacentric with polymorphism in the size of its short arms and the Y chromosome was a very small acrocentric. Autosomal pairs 1, 10, 19, 20 and 21, the X and the Y chromosomes were the only identifiable chromosomes after conventional Giemsa staining (Fig. 2).

**Figure 3.**
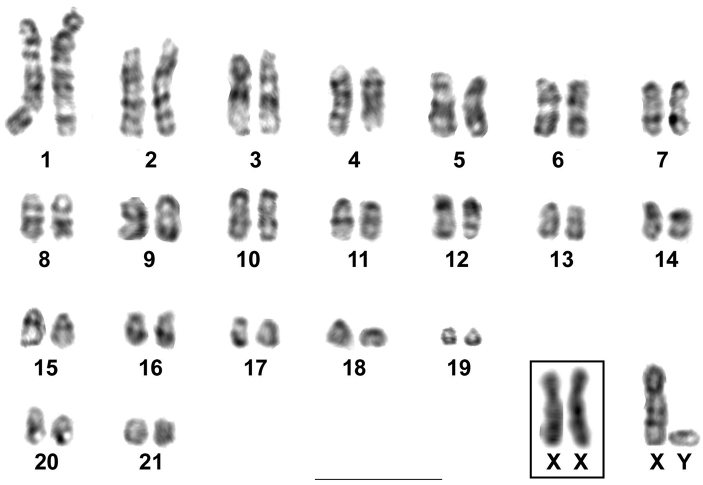
GTG-banded karyotype of *Rhipidomys* sp. male (2n=44, FN=48). In the inset, the sex chromosomes of a female. Bar = 10 µm.

After GTG-banding all the autosomes and the sex chromosomes could be identified. The X chromosome presented the two typical mammalian dark GTG-bands in its long arm and no bands were observed on its short arms. The Y chromosome had an indistinct staining (Fig. 3).

**Figure 4.**
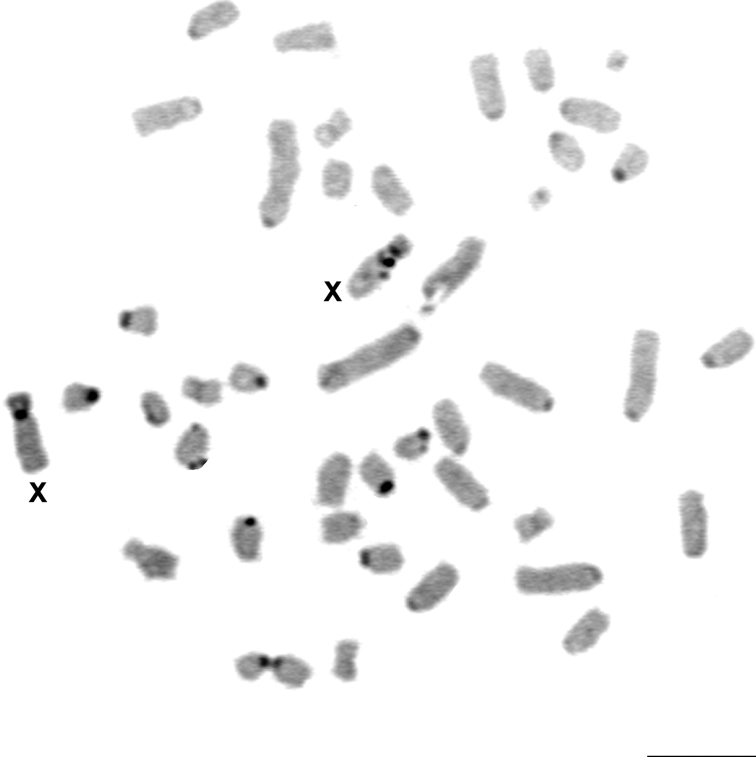
CBG-banding in a metaphase of *Rhipidomys* sp. female (2n=44, FN=48). Bar = 10 µm.

CBG-banding revealed the presence of constitutive heterochromatin in the pericentromeric region of most autosomal pairs. The short arm of the X chromosome was entirely heterochromatic with a stronger stained pericentromeric region and the Y chromosome displayed a small pericentromeric C-band (Fig. 4).

**Figure 5.**
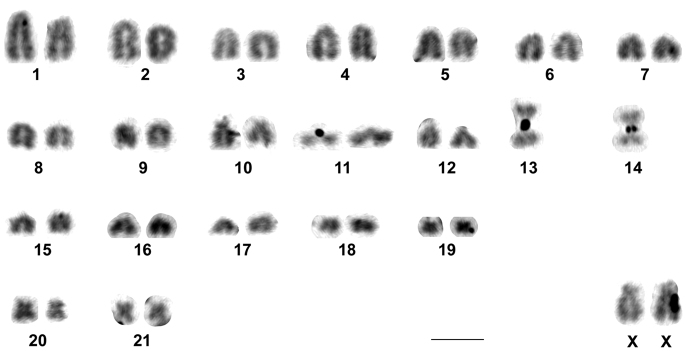
Silver staining of the nucleolus organizer regions (Ag-NORs) in the karyotype of *Rhipidomys* sp. female (2n=44, FN=48). Bar = 10 µm.

**Figure 6.**
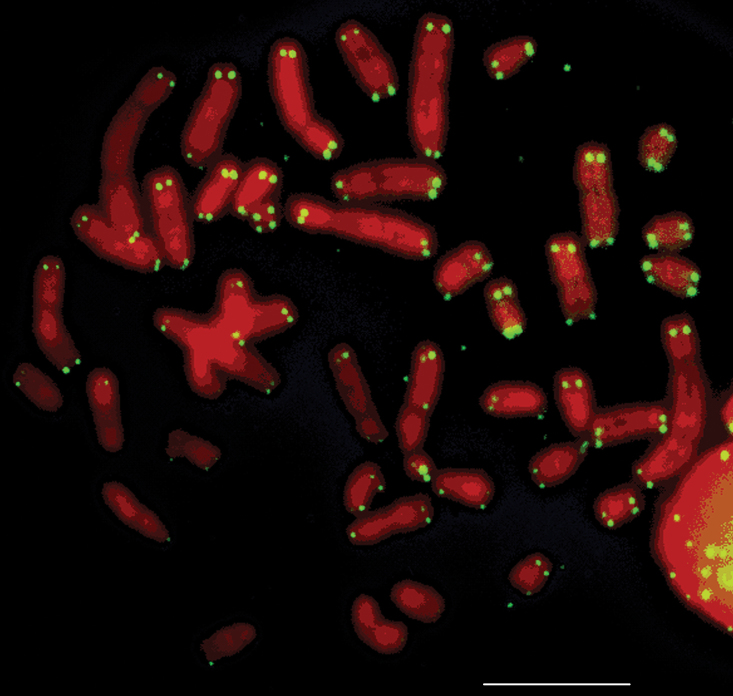
Metaphase of *Rhipidomys* sp. female (2n=44, FN=48) after FISH with a telomere probe. Bar = 10 µm.

Silver staining revealed one to five nucleolus organizer regions (Ag-NORs) per cell. The Ag-NORs were located on the short arms of medium/small acrocentric autosomes. From the 151 analyzed cells, the majority (57) showed four Ag-NORs. Associations between NORs were frequent (Table 2, Fig. 5). FISH with the telomere sequences revealed signals only at the telomere regions of all chromosomes (Fig. 6).

**Table 2.** Number of Ag-NORs per cell in *Rhipidomys* sp. (2n=44, FN=48).

		**Number of chromosomes with Ag-NORs**					
		**1**	**2**	**3**	**4**	**5**	**Total**
Number of cells	MCNM 1643 (Male)	4	5	10	10	1	30
	MCNM 1644 (Male)	2	6	6	13	3	30
	MCNM 1646 (Female)	5	4	10	7	4	30
	MCNM 1647 (Female)	1	3	16	11	0	31
	MCNM 1648 (Female)	1	0	12	16	1	29
	Total	13	18	54	57	9	151

## Discussion

Besides the karyotype of *Rhipidomys* sp. presented herein, four other species of *Rhipidomys* with the karyotype formula of 2n=44 and FN=48 have been described: *Rhipidomys latimanus* Tomes, 1860 from Colombia, for which no karyotype picture has been presented (Gardner and Patton 1976); *Rhipidomys macrurus* from the Brazilian states of Goiás and Bahia (Svartman and Almeida 1993, Pereira and Geise 2007), *Rhipidomys leucodactylus* collected in the Brazilian states of Rondônia and Goiás (Zanchin et al. 1992, Andrades-Miranda et al. 2002) and in Venezuela (Aguilera et al. 1994), and *Rhipidomys itoan* from the states of São Paulo and Rio de Janeiro. The animals from Venezuela were originally identified as *Rhipidomys sclateri* (Aguilera et al. 1994), which was later recognized as a synonym of *Rhipidomys leucodactylus* (Musser and Carleton 2005).

The karyotype of *Rhipidomys* sp. studied herein differed from that of *Rhipidomys macrurus* (2n=44, FN=48) from Goiás (Svartman and Almeida 1993) in the morphologies of pair 10 and of the smallest autosome pair. Pair 10 was subtelocentric in *Rhipidomys* sp. and acrocentric in *Rhipidomys macrurus*, whereas the smallest autosome pair was acrocentric in *Rhipidomys* sp. and metacentric in *Rhipidomys macrurus*. The X chromosome was submetacentric in *Rhipidomys cariri* and acrocentric in *Rhipidomys macrurus*. The CBG-banding patterns also differed between both species, as only a very small amount of constitutive heterochromatin was detected in *Rhipidomys macrurus* (Svartman and Almeida 1993), compared to the pericentromeric C-bands found in most autosomes of *Rhipidomys* sp. (Fig. 3). Silver staining revealed the presence of 1 to 5 chromosomes with nucleolus organizer regions (Ag-NORs) in *Rhipidomys* sp. All the NOR-bearing chromosomes were medium acrocentrics similar to the three pairs that presented Ag-NORs in *Rhipidomys macrurus* (Svartman and Almeida 1993).

GTG-banding patterns have not been described for *Rhipidomys leucodactylus*, also with 2n=44 and FN=48. From the three biarmed autosomes found in the karyotype of this species, two are comparable in size to the medium acrocentric pair 15 and the third is the smallest autosome pair (Zanchin et al. 1992, Aguilera et al. 1994, Andrades-Miranda et al. 2002), whereas in *Rhipidomys* sp. the biarmed chromosomes correspond in size to pairs 10, 15 and 16. The X chromosome also differed between both species, being biarmed in *Rhipidomys* sp. and acrocentric in *Rhipidomys leucodactylus*. Interestingly, the complement of *Rhipidomys leucodactylus* seemed identical to that of *Rhipidomys macrurus* from Goiás (Svartman and Almeida 1993), but the absence of GTG-banding patterns of *Rhipidomys leucodactylus* in the literature hindered further comparisons.

In *Rhipidomys itoan* with 2n=44 and FN=48 the smallest autosome pair was a submetacentric (Costa et al. 2011), differing from the acrocentric smallest autosome of *Rhipidomys* sp. presented herein. Morphological variations were observed in two large pairs of *Rhipidomys itoan*, that could be acrocentric or biarmed, leading to higher fundamental numbers (FN=49 and 50). No such variation was detected in our specimens.

The absence of banding patterns descriptions limited the comparisons of the complement of *R.* sp. described in this work and those of *Rhipidomys itoan* and *Rhipidomys leucodactylus* to conventionally stained chromosomes.

The *Rhipidomys* species already recorded in Minas Gerais were *Rhipidomys macrurus*, which is probably distributed in the remaining Cerrado fragments of the state, *Rhipidomys mastacalis*, which was collected in the Atlantic Forest in eastern and southern Minas Gerais, and *Rhipidomys tribei*, known from only a few sites in the southeastern part of Minas Gerais (Tribe 1996, Bonvicino et al. 2008; Costa et al. 2011).

*Rhipidomys mastacalis* is characterized by a high fundamental number (FN=74 through 80) (Zanchin et al. 1992, Paresque et al. 1994, Tribe 1996, Andrades-Miranda et al. 2002) which promptly allows to differentiate its karyotype from that of *Rhipidomys* sp. (FN=48). On the other hand, *Rhipidomys macrurus* (FN=48-50; variation due to polymorphism in the morphology of pair 10) (Svartman and Almeida 1993, Pereira and Geise 2007) presented a complement very similar to that of *Rhipidomys* sp. (FN=48). Nevertheless, as discussed above, the two karyotypes differ in the morphology of the smallest autosome pair (pair 19, acrocentric in *Rhipidomys* sp., and pair 21, metacentric in *Rhipidomys macrurus*), and in the amount of constitutive heterochromatin, which can thus be used to differentiate both species. No chromosome data are available for *Rhipidomys tribei*.

FISH with telomere sequences has been previously performed in specimens of *Rhipidomys nitela*, *Rhipidomys mastacalis* and *Rhipidomys leucodactylus* (Andrades-Miranda et al. 2002), *Rhipidomys* prope *mastacalis* and in animals of two unidentified species (Silva and Yonenaga-Yassuda 1999). As for *Rhipidomys* sp. presented herein, only terminal signals were observed in the cells of all these specimens. Interstitial signals, which could give clues on chromosome rearrangements, have not been observed in *Rhipidomys* as yet.

The identification of *Rhipidomys* specimens from southeastern Brazil at the species-level has proven to be specially challenging, with *Rhipidomys macrurus* and *Rhipidomys mastacalis* being among the most taxonomically complicated taxa studied (Musser and Carleton 2005; Tribe 1996, Costa et al. 2011).

Chromosome analyses may be useful for the identification of species, especially in complicated taxonomic groups, as is the case of many rodent taxa. As stressed by Tribe (1996), the use of non-morphological characters, as karyotypes, allozymes and DNA sequences, may help in clarifying the phylogenetic relationships and in the taxonomic identification of *Rhipidomys* species. This prediction has proven right in works like those of Costa et al. (2011) and Rocha et al. (2011), which used molecular data to further the knowledge of the genus, resulting in the description of new species and in the clarification of some phylogenetic relationships. Likewise, karyotypical data, especially those including banding patterns, are likely to add new information and to help in clarifying the taxonomy and phylogenetics of this intriguing rodent genus.

With the available data, it seems evident that a larger collection effort including a wider geographical range and complemented by cytogenetic and molecular studies will be needed in order to establish the phylogenetic relationships and phylogeography of *Rhipidomy*s in Brazil. Nevertheless, as exemplified in this work, the use of chromosome data has already proven to be a useful tool in resolving taxonomic issues in this genus.
